# Evaluating the Global Digital Impact of Psoriasis Action Month and World Psoriasis Day: Serial Cross-Sectional Study

**DOI:** 10.2196/49399

**Published:** 2023-09-04

**Authors:** Kashish Malhotra, Pranshul Aggarwal, Sakshi Malhotra, Kashish Goyal

**Affiliations:** 1 Dayanand Medical College and Hospital Ludhiana India; 2 Pandit Bhagwat Dayal Sharma Post Graduate Institute of Medical Sciences Rohtak India

**Keywords:** psoriasis, psoriasis awareness, social media, digital health, Psoriasis Action Day, World Psoriasis Day, skin, dermatology, awareness, health promotion, trends, Twitter, tweets, cross-sectional

## Introduction

Psoriasis is one of the most common chronic inflammatory skin disorders, affecting over 100 million people globally [1]. The chronic nature of psoriasis contributes to its overall burden, leading to a significant psychological and social impact on those who are affected [2]. Hence, with the objective of educating, dismissing stereotypes, and providing information about timely management by involving the global community, Psoriasis Action Month (previously Psoriasis Awareness Month) in August and World Psoriasis Day on October 29 are recognized worldwide. Social media platforms have developed into powerful information sources that influence opinions about medicine in a variety of ways [3]. We evaluated the digital impact of Psoriasis Action Month and World Psoriasis Day by studying trends in total tweets posted, identifying top keywords, understanding collaborations via a network analysis, and studying the global reach of these awareness initiatives to steer future policy development.

## Methods

In this serial cross-sectional analysis, we used broad search queries to study the total number of tweets posted from 2014 to 2022 targeting psoriasis, Psoriasis Action Month, and World Psoriasis Day via Sprout Social. Being consistent with previous studies [4-7], a social network analysis using the ForceAtlas2 model was conducted to contextualize the tweets via Socioviz. Each entity was depicted as a node with a size set proportional to its frequency in tweets. Similar colors depicted sets of arguments that are frequently associated together. Collaboration proportion was studied using connections between users. Beyond social media, we analyzed Google Trends relative search volume data by region from 2014 to 2022 to gauge interest in Psoriasis Action Month and World Psoriasis Day without any language or geographic restrictions.

## Results

**Figure 1:**
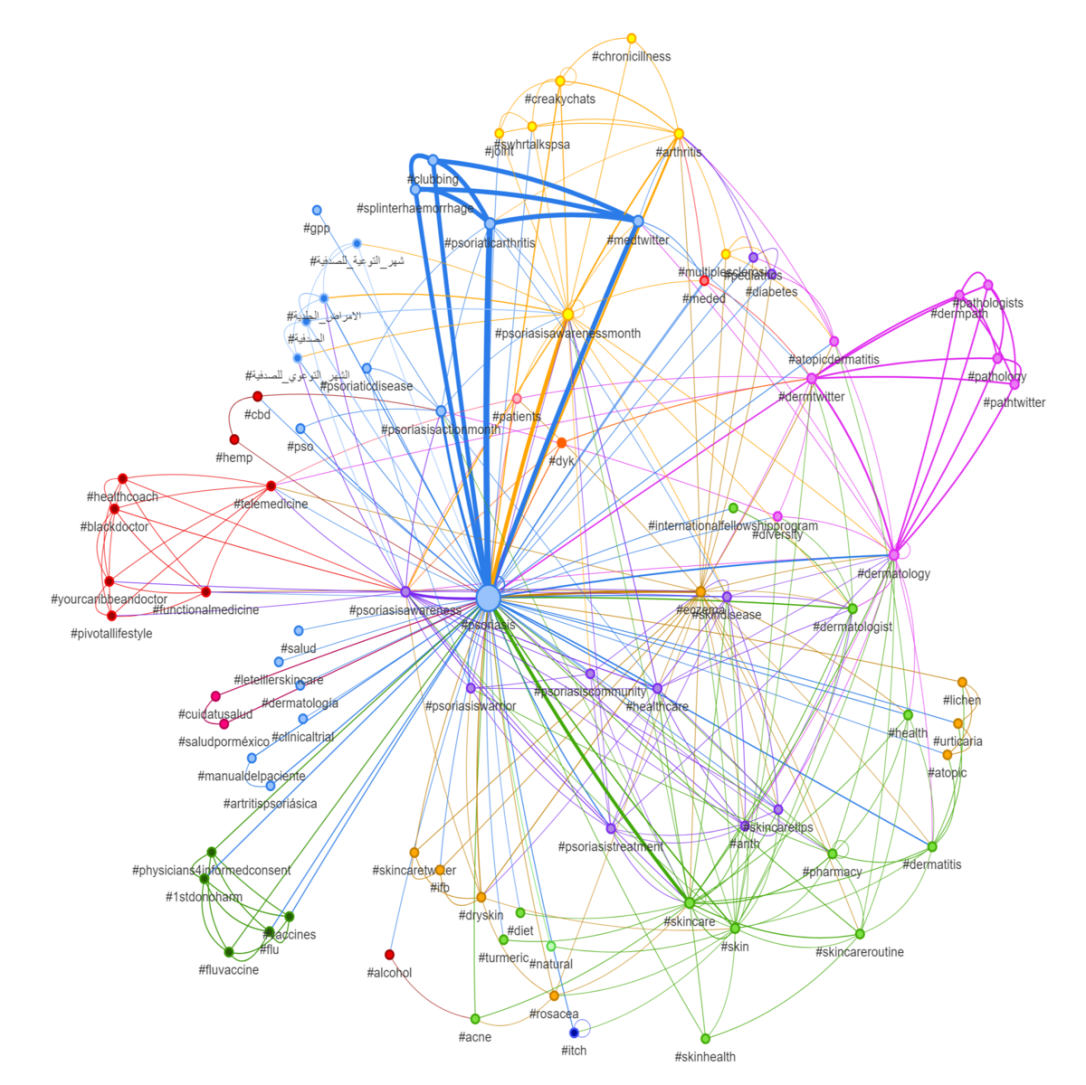
Social network analysis of tweets related to psoriasis during Psoriasis Action Month using the ForceAtlas2 model (search query: psoriasis OR #psoriasis).

A total of 3384 and 1925 tweets were identified in 2022 related to Psoriasis Action Month and World Psoriasis Day, respectively, during the specified time period, showing a change of +67.68% and –15.75%, respectively, when compared to total tweets in 2021 (Table 1). Tweets related to Psoriasis Action Month and World Psoriasis Day constituted 7.99% and 31.18%, respectively, of the total tweets posted about psoriasis during their respective time range in 2022. Yearly trends in the total tweets about Psoriasis Action Month showed a plateau over the last few years, but volumes have now increased. On the other hand, tweets on World Psoriasis Day were previously increasing annually but have now declined in a somewhat cyclical way. Interestingly, more users tweeted about World Psoriasis Day than Psoriasis Action Month between 2019 and 2021. The network analysis showed that the collaboration proportion among the top 100 influencers was 42%. The most frequently associated keywords were “fries,” “eczema,” “butter,” “scrub,” and “health.” The most frequently associated hashtags were “#psoriaticarthritis,” “#psoriasisawarenessmonth,” “#medtwitter,” “#splinterhemorrhage,” and “#clubbing.” Additional themes linked to other skin diseases and chronic illnesses, skincare, and management options were identified (Figure 1). The Google Trends analysis demonstrated that the majority of web searches were from the American, European, Russian, South Asian, and Australian regions with limited involvement from African and Central Asian countries.

**Table 1:** Tweets posted about psoriasis, Psoriasis Action Month, and World Psoriasis Day from 2014 to 2022.

Year	Total tweets about Psoriasis Action Month (August 1-31; search query 1^a^)	Total tweets about psoriasis (August 1-31; search query 2^b^)	Proportion^c^ (%)	Total tweets about World Psoriasis Day (October 28-30; search query 3^d^)	Total tweets about psoriasis during (October 28-30; search query 2)	Proportion^e^ (%)
2014	995	15,709	6.33	1827	9466	19.30
2015	1420	23,884	5.95	2614	12,532	20.86
2016	1905	20,063	9.50	1739	10,840	16.04
2017	1684	16,416	10.26	1503	7263	20.69
2018	2363	23,228	10.17	2203	7159	30.77
2019	2342	61,354	3.82	2363	8466	27.91
2020	1882	32,574	5.78	2977	8435	35.29
2021	2023	23,067	8.77	2285	6333	36.08
2022	3384	42,353	7.99	1925	6174	31.18

^a^Search query 1: psoriasis awareness month OR #psoriasisawarenessmonth OR psoriasis month OR #psoriasismonth OR psoriasis action month OR #psoriasisactionmonth OR psoriasis awareness OR #psoriasisawareness.

^b^Search query 2: psoriasis OR #psoriasis.

^c^Proportion was calculated as search query 1/search query 2 × 100.

^d^Search query 3: world psoriasis day OR #worldpsoriasisday OR psoriasis awareness day OR #psoriasisawarenessday OR psoriasis day OR #psoriasisday OR psoriasis awareness OR #psoriasisawareness.

^e^Proportion was calculated as search query 3/search query 2 × 100.

## Discussion

The limited presence of psoriasis-related content on digital media platforms highlights a missed opportunity to effectively engage a wider audience and disseminate accurate information about psoriasis [8]. Our ballpark estimates highlight the need to increase collaboration, targeted efforts, and partnerships among low-, middle-, and high-income countries especially in Africa, as shown in previous publications [9,10]. Because of the greater cumulative reach, one could naturally assume that an awareness month will have a greater impact than a 1-day campaign. However, the case of World Psoriasis Day recently amassing more reach than Psoriasis Action Month needs to be further studied, and constructive feedback should be implemented for other similar events. More emphasis needs to be given to prioritizing event-specific hashtags and promoting collaboration in digital toolkits to amplify messages on social media, which may also later help to assess impact and plan ahead. 

The unavailability of data sets from other social media platforms, such as Facebook, Instagram, and TikTok, due to a lack of access to their application programming interface is a limitation of this study. Furthermore, Google Trends provides only relative search volume data, which are not equivalent to absolute values. It is crucial to study linked keywords and hashtags within the appropriate context as these could represent noise, off-topic posts, or misclassifications as seen in the associated hashtag “#fries.” Considering the dynamic nature of online media, statistical techniques such as controlled experiments, data normalization, and segmented analysis are needed in future large-scale studies to mitigate platform-induced biases and study user behavior shifts to collect detailed insights.

## Data Availability

The data presented in this study are included in the paper. Further inquiries can be directed to the corresponding author.
